# Mobile Access to ClinicalConnect: A User Feedback Survey on Usability, Productivity, and Quality

**DOI:** 10.2196/mhealth.4011

**Published:** 2015-04-14

**Authors:** Bell Raj Eapen, Barbara Chapman

**Affiliations:** ^1^HNHB eHealth OfficeHamilton, ONCanada

**Keywords:** mHealth, health information exchange, ClinicalConnect

## Abstract

**Background:**

ClinicalConnect, a federated clinical viewer for South West Ontario, Canada, launched a mobile interface in June 2012.

**Objective:**

The aim of the study was to assess usability of the mobile interface and the perceived impact on productivity of health care providers and quality of healthcare delivery.

**Methods:**

A survey was conducted using the System Usability Scale (SUS) and questionnaires designed to measure productivity and quality based on Canada Health Infoway's Benefits Evaluation framework.

**Results:**

The mean SUS score was 67 based on 77 responses. The mean scores for productivity and quality were 3.37 (N=74) and 3.62 (N=71), respectively, on a 5-point Likert scale where 3 was neutral.

**Conclusions:**

Users perceived the mobile interface of ClinicalConnect as useful but were neutral about the ease of use.

## Introduction

Mobile devices are rapidly becoming a part of everyday life as both communication and information tools. Recent studies indicate that this is true in the case of health care providers as well. Health care providers often use their mobile devices to access educational material, pharmaceutical compendiums, practice guidelines, clinical pathways, and electronic medical records [1].

Electronic health information exchange (HIE), in which patients’ clinical data is efficiently shared between care delivery settings, is expected to produce a number of quality improvements and cost savings [2]. ClinicalConnect is a federated clinical viewer that provides health information exchange between health services and care providers of South West Ontario [3]. It is considered to be one of the largest federated HIE models in North America. A mobile version of ClinicalConnect was developed and deployed in June 2012.

Health care providers often fail to realize the intended effects of their eHealth systems due to inadequate usability [4]. Despite the widely held belief that the computerization of health information systems contributes to improved quality of patient care and care management, studies have found medical staff attitudes toward computerization to be negative [5]. In order to discern whether the ClinicalConnect mobile version was meeting user requirements, an evaluation survey was conducted in November 2013. A range of survey tools was used for the assessment with a focus on usability, and the perceived impact of the mobile version on the quality of patient care and productivity of health care providers.

## Methods

### Tools

The System Usability Scale (SUS), a nonproprietary validated survey tool [6], was used to assess the usability of the ClinicalConnect mobile interface. SUS includes 10 statements presented on a 5-point Likert scale, which results in an overall score from 0 to 100 that indicates the perceived usability of the interface.

Canada Health Infoway published a Benefits Evaluation Indicators Technical Report in 2006 that was subsequently updated in 2012 to provide guidance for benefits evaluation planning related to information and communications technology (ICT) in health care [7]. The framework proposes the indicators that could have an impact on productivity and quality of health care. Based on these indicators, productivity and quality questionnaires using a 5-point Likert scale [8] were designed. Productivity and quality questionnaires had 8 (Table 1) and 10 questions (Table 2), respectively.

The SUS, productivity, and quality survey tools were hosted and administered using SurveyMonkey [9].

### Participants

Five hundred and four health care providers who expressed interest in participating in periodic evaluations while signing up for the HIE service were sent an e-mail with a link to the survey. A second reminder e-mail was sent if no response was received from a provider. One hundred and ten providers responded to the survey, though some surveys were incomplete. Seventy-seven responses were obtained for SUS. On productivity and quality scores, we received 74 and 71 responses, respectively.

### Analysis

Results of the SUS questionnaire were recoded and normalized, and the mean SUS score and the standard deviation were then recorded. The Likert-type questions for productivity and quality were independently tabulated. Since all questions within each category measure a single concept, the values were combined into a composite score by calculating the mean and standard deviation. Scores for negatively worded questions (fifth question in the productivity matrix and second and third questions in the quality matrix) were normalized prior to the calculation of the composite score.

## Results

Eighty-five (77%) of the respondents were physicians. The mean SUS score was 67 (SD 14.4) with a percentile score of 46.9 [10]. This means that the mobile version of ClinicalConnect can be considered more usable than 46.9% of all products evaluated with the SUS instrument.

Table 1 depicts the responses to the productivity-related questions and Table 2 summarizes the quality questions. The mean productivity score was 3.37 (SD 1.06) and the mean quality score was 3.62 (SD 0.99), with 3 being neutral on the 5-point Likert scale.

**Table 1:** Productivity questions and summary of responses (N=74).

Answer options	Strongly agree	Agree	Neither agree nor disagree	Disagree	Strongly disagree
	n (%)	n (%)	n (%)	n (%)	n (%)
Accessing patient/client test results and information on my mobile device has decreased time spent tracking down or waiting for these reports	15(20)	27(36)	17(23)	13(18)	2(3)
Using a mobile device allows me to spend more face-to-face time with my patient/client	5(7)	19(26)	31(42)	18(24)	1(1)
I have more portability as I can now access ClinicalConnect with a mobile device	19(26)	47(64)	4(5)	3(4)	1(1)
The “New Results” feature draws my attention to results quickly	10(14)	34(46)	22(30)	6(8)	2(3)
It is difficult to view patient information on my mobile device screen	6(8)	21(28)	22(30)	21(28)	4(5)
I can access information faster on my mobile device than on a desktop computer	3(4)	18(24)	20(27)	17(23)	16(22)
Treatment decisions are made faster now that I can access patient/client information on my mobile device	8(11)	20(27)	32(43)	14(19)	0(0)
Access to patient information on my mobile device allows for better communication between health care providers	14(19)	31(42)	22(30)	7(9)	0(0)

**Table 2:** Quality questions and summary of responses (N=71).

Answer options	Strongly agree	agree	Neither agree nor disagree	Disagree	Strongly disagree
	n (%)	n (%)	n (%)	n (%)	n (%)
Education activities are enhanced when the patient/client can view their results on my mobile device with me	7(10)	16(23)	33(46)	14(20)	1(1)
There is high risk that a mobile device can be the source of a nosocomial infection	3(4)	11(15)	24(34)	24(34)	9(13)
Current results are not easy to access on a mobile device	5(7)	16(23)	16(23)	25(35)	9(13)
A mobile device allows faster access to vital patient information facilitating quicker consultation, diagnostic tests, and interventions	14(20)	29(41)	17(24)	11(15)	0(0)
Access to patient/client information anywhere, anytime enhances consultations, referrals, and handoffs	17(24)	38(54)	10(14)	5(7)	1(1)
I am more confident that my mobile device is cleaner than the desktop computer that is used by multiple people	11(15)	24(34)	23(32)	8(11)	5(7)
I can better prioritize my actions to follow-up on test results with the “New Results” feature	6(8)	29(41)	31(44)	4(6)	1(1)
I feel more confident in care decisions because I have the information I need at my fingertips	15(21)	30(42)	22(31)	3(4)	1(1)
I am less likely to order a duplicate test because I have easier access to current results	15(21)	36(51)	16(23)	3(4)	1(1)
HCPs should have access to their patient’s/client’s health information no matter where they are	33(46)	29(41)	7(10)	2(3)	0(0)

## Discussion

HIE is the process of sharing electronic health information between different providers and organizations. As in most other health information systems, mobile devices are increasingly becoming popular as an HIE platform [11].

The SUS consists of 10 alternating positively and negatively worded statements scored on a 5-point Likert scale. The mean SUS score for 2324 surveys about usability conducted over a 10-year period was 70.14 with a median score of 75 [6].

Inadequate usability is a major cause of failure of eHealth systems [4], especially of mobile platforms. Our data show that health service providers perceive the mobile interface of ClinicalConnect as useful, but are neutral about the ease of use. This pattern has been noticed before in other mHealth interventions [12]. The ease of use can be affected by factors beyond mobile user interface such as ergonomic and social aspects [13].

HIE is vital for improving efficiency and quality of health care. It has been demonstrated that perceived usefulness is a stronger predictor of the use of an eHealth technology than the perceived ease of use [14]. Most of our respondents agreed that ClinicalConnect on mobile devices had a positive impact on their productivity and quality. Anywhere, anytime access to patient information on mobile devices was perceived as an important factor in faster consultations, referrals, and handoffs, and in improved communication between health care providers. However, information access on mobile devices was not considered faster than the desktop counterpart, and there was no consensus on whether mobile devices allow more face-to-face time with patients. This corroborates previous studies that showed that physicians consider improvements in the quality domain as the overarching benefit of HIE [15].

Though HIE programs have demonstrated clinical value in some situations such as emergency departments [16], a consistent empirical proof of value is lacking [17]. In a study, emergency physicians reported workflow disruptions from HIE use [18]. Our study suggests that mobile access to integrated health records is perceived as beneficial, especially to the quality of patient care. However, our sample size may be insufficient and not representative enough of the health care roles for us to statistically draw a precise conclusion.

It is important for HIE system implementations to be integrated into the care practice improvement process [4]. The study shows that bringing data from disparate health care systems to the point of care via mobile HIE systems has a perceived potential for improvements in patient care.

## Abbreviations

HIE: health information exchange
HNHB: Hamilton Niagara Haldimand Brant
ICT: information and communications technology
LHIN: local health integration network
SUS: System Usability Scale
